# Prevalence of Bodily Distress Syndrome and Prediction of Patient Outcomes: Cohort Study of 3762 Individuals With Persistent Pain

**DOI:** 10.1002/ejp.70212

**Published:** 2026-01-20

**Authors:** Live Landmark, Hans Fredrik Sunde, Egil A. Fors, Leif Edward Ottesen Kennair, Silje Endresen Reme

**Affiliations:** ^1^ Department of Psychology, Faculty of Social and Educational Sciences Norwegian University of Science and Technology Trondheim Norway; ^2^ The Mind‐Body Lab, Department of Psychology, Faculty of Social Sciences University of Oslo Oslo Norway; ^3^ Centre for Fertility and Health Norwegian Institute of Public Health Oslo PO Norway; ^4^ Department of Public Health and Nursing, Faculty of Medicine and Health Sciences Norwegian University of Science and Technology Trondheim Norway; ^5^ Department of Pain Management and Research Oslo University Hospital Oslo Norway

## Abstract

**Background:**

Persistent physical symptoms are common and often result in disability and high healthcare use. To capture how such symptoms co‐occur, Bodily Distress Syndrome (BDS, also called Functional Somatic Disorders) was developed as an empirically derived construct, in contrast to consensus‐based syndromes such as fibromyalgia and chronic fatigue syndrome/myalgic encephalomyelitis (CFS/ME). BDS is distinct from ICD‐11 Bodily Distress Disorder and reflects the multisystem symptom pattern described in the Functional Somatic Disorder framework. However, its prevalence, symptom structure and prognostic relevance in pain populations have not been mapped.

**Methods:**

This study included 3762 individuals referred to a tertiary pain clinic. At baseline, participants reported standardised measures of fatigue, insomnia, pain catastrophizing, psychological distress, perceived injustice, health‐related quality of life and disability. After 12 months, they reported disability and perceived change. BDS severity was classified from predefined symptom cluster criteria. We examined prevalence, clinical correlates and prognostic utility.

**Results:**

92.5% met the criteria for moderate or severe BDS. They reported more severe physical symptoms, elevated psychological distress and reduced functioning than those not meeting the criteria. Severe BDS was more common among women, those without higher education and individuals outside the workforce. After 12 months, individuals with BDS showed less improvement in functioning and reported lower perceived treatment benefit.

**Conclusion:**

BDS was common in this outpatient hospital cohort and may offer a clinically useful lens for capturing multisystem complexity in specialised pain services. Incorporating BDS screening into routine assessment could help identify individuals with complex symptoms and support more mechanism‐oriented treatment approaches.

**Significance Statement:**

Bodily Distress Syndrome (BDS) is highly prevalent in tertiary pain care and linked to more severe symptoms, psychosocial burden and poorer long‐term outcomes. The findings support BDS as a clinically useful framework for identifying patients with complex symptom profiles and for guiding interdisciplinary, mechanism‐oriented approaches to pain management.

## 1 Introduction

Persistent physical symptoms (Löwe et al. 2024), including pain, fatigue, and insomnia, are common (Goldberg and McGee 2011; Morin and Jarrin 2022; van't Leven et al. 2009). In Norway, 31% of adults report chronic pain (Landmark et al. 2013), 22% fatigue (Loge et al. 1998), and 21.7% insomnia (Sivertsen et al. 2021). These symptoms frequently co‐occur (Hiestand et al. 2023) and substantially contribute to disability and healthcare costs (Gaskin and Richard 2011; Reynolds et al. 2004; Reynolds and Ebben 2017).

The classification of these symptoms remains debated. ‘Splitters’ favour separate diagnoses (Galvez‐Sánchez and Reyes del Paso 2020; National Institute for Health and Care Excellence 2021b), whereas ‘lumpers’ propose broader syndromic constructs (Burton et al. 2020; Henningsen, Zipfel, et al. 2018). This debate is reflected in ICD‐11, where fibromyalgia and chronic fatigue syndrome/myalgic encephalomyelitis (CFS/ME) remain separate conditions (World Health Organization 2022). Both involve fatigue, cognitive impairments, and unrefreshing sleep (Ramírez‐Morales et al. 2022). However, fibromyalgia requires widespread pain (Wolfe et al. 2016), whereas CFS/ME is defined by post‐exertional malaise (PEM) (National Institute for Health and Care Excellence 2021b). These symptoms also frequently co‐occur (Barhorst et al. 2022), and PEM (May et al. 2020), pain (Shaw et al. 2016), disturbed sleep (Sivertsen et al. 2009) and fatigue (Chalder et al. 2010) are each associated with psychological distress. Assessment is further complicated by the absence of biomarkers (Lacourt et al. 2013). In line with this nosological split, clinical guidelines also differ, with recommendations for activity in fibromyalgia (Macfarlane et al. 2017; National Institute for Health and Care Excellence 2021a) and for energy conservation in CFS/ME (National Institute for Health and Care Excellence 2021b).

Bodily Distress Syndrome (BDS) has been proposed as a unifying framework for these symptom patterns (Fink et al. 2007). The construct was empirically derived from clinical samples (Fink et al. 2007) and defined by symptom clusters across multiple organ systems (Fink and Schröder 2010). BDS is distinct from the ICD‐11 Bodily Distress Disorder, which emphasises psychobehavioral responses (Henningsen and Löwe 2025), and aligns with broader conceptual development such as the Functional Somatic Disorder framework (Burton et al. 2020; Gormsen et al. 2025).

BDS shows a female predominance similar to fibromyalgia and CFS/ME (Bakken et al. 2014; Clauw 2014), with population prevalence estimates between 11.8% and 16.1% (Häuser et al. 2020; Petersen, Schröder, et al. 2020). The construct has been examined in adolescents (Münker et al. 2022), but not in chronic pain populations.

In a previous study using the same clinical sample as the present study (Landmark et al. 2024), we found substantial levels of fatigue, pain, and insomnia. Based on these findings, we expected a high prevalence of BDS, greater disability among affected individuals in line with previous findings (Ma et al. 2022), and less improvement over time (Budtz‐Lilly, Vestergaard, et al. 2015).

We aimed to:

1. Estimate the prevalence of BDS in a tertiary outpatient pain clinic.
2. Characterise individuals who met BDS criteria.
3. Assess whether BDS at baseline predicted pain‐related disability and perceived change at 12‐month.

## 2 Methods

### 2.1 Sample

This study draws on longitudinal data from 4288 individuals in the Oslo University Hospital Pain Registry (OPR) (Granan et al. 2019), of whom 3762 had valid BDS data and were included. The clinic is a tertiary referral centre in Southeastern Norway, receiving ~1000 new referrals annually for multidisciplinary pain management. The cohort includes individuals with diverse persistent pain conditions, mainly non‐malignant conditions.

We previously used OPR data to study psychosocial predictors of pain‐related disability (Landmark et al. 2024). Here, we examine the classification and prognostic value of BDS, including new longitudinal outcomes. BDS was assessed with the BDS checklist (Budtz‐Lilly, Fink, et al. 2015) as a case‐finding instrument to identify probable BDS based on symptom patterns. The dataset lacked ICD‐coded pain diagnoses, medical comorbidities and psychiatric diagnoses and therefore did not provide information needed to characterise pain subtypes, report comorbidity profile or perform clinical differential diagnostic assessment. Because differential diagnostic exclusion cannot be performed in this registry design, the BDS checklist was used as a symptom‐based case‐finding instrument rather than a diagnostic tool.

**FIGURE 1:**
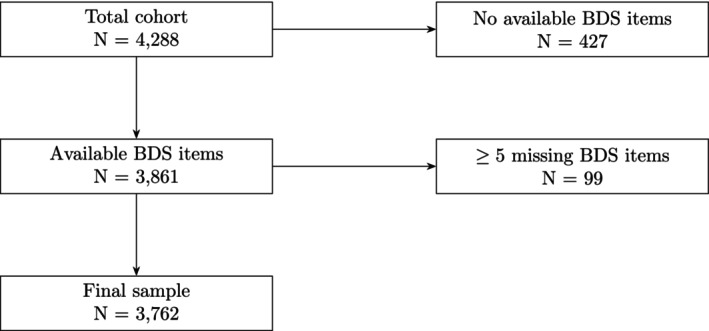
Flowchart of participant inclusion and exclusion. From the total cohort (*N* = 4288), individuals with fully missing BDS data (*n* = 427) and those with excessively many missing items (*n* = 99) were excluded. The final sample used in BDS‐related analyses comprised 3762 individuals.

Of the 4288 individuals, 427 had missing data on all BDS items and were excluded (Figure 1). Another 545 had partial BDS data, and 99 with ≥ 5 missing items were excluded. For the remaining 446 individuals with ≤ 4 missing items, missing responses were coded as ‘symptom not present’ (0), which may slightly underestimate prevalence and severity. The final sample comprised 3762 individuals (87.7% of the cohort).

### 2.2 Data Collection

Data were collected from October 2015 to November 2020 via standardised electronic questionnaires completed before consultation. Some individuals could not complete the full version, for example if they were under 18 years or required an interpreter. The shorter version did not include the BDS checklist, so these individuals were not included.

Over 90% of referred individuals completed baseline questionnaires. Only those providing written informed consent (~80%) were included in the OPR research database. Consenting patients received an encrypted email link to follow‐up questionnaires at 12 and 36 months; the present study uses baseline and 12‐month data.

### 2.3 Measures

#### 2.3.1 Sociodemographic and Clinical Characteristics

We included age (years), gender (male/female), education, employment status (working, student, military service or not working), and pain duration (years and months).

#### 2.3.2 Bodily Distress Syndrome

We applied the 25‐item BDS checklist (Budtz‐Lilly, Fink, et al. 2015), covering four symptom clusters: cardiopulmonary, gastrointestinal, musculoskeletal, and general. Participants were instructed: ‘During the last four weeks, have you been bothered by…’ followed by 25 symptoms rated on a 5‐point scale: 0 (Not at all) to 4 (A lot). Responses were pre‐dichotomized as in the original operationalization: scores 0–1 coded as 0 (symptom absent), scores 2–4 coded as 1 (symptom present).

For categorical classification, we followed the original case‐finding algorithm (Budtz‐Lilly, Fink, et al. 2015), based on five binary indicators: ≥ 4 symptoms in each of the four clusters and ≥ 4 symptoms overall.

1. ≥ 4 symptoms in the cardiopulmonary cluster
2. ≥ 4 symptoms in the gastrointestinal cluster
3. ≥ 4 symptoms in the musculoskeletal cluster
4. ≥ 4 symptoms in the general symptoms cluster, and
5. ≥ 4 symptoms overall

Moderate BDS = criteria met in 1–3 indicators; severe BDS = criteria met in 4–5 indicators (Budtz‐Lilly, Fink, et al. 2015). To capture dimensional variation and address research questions 2 and 3, we included a continuous BDS sum score (0–25), reflecting the total number of reported symptoms (Petersen, Rosendal, et al. 2020). The BDS checklist is reliable and valid (Wertenbruch‐Rocke et al. 2021). In our sample, Cronbach's α was 0.866, reported for descriptive purposes only, as the four distinct clusters are intended to assess different symptom domains.

A Norwegian version of the BDS checklist has been used in clinical practice at Oslo University Hospital (Oslo University Hospital 2020). The items were translated from Danish by a senior consultant with a doctoral degree and extensive clinical experience in the field. The translation was reviewed with support from a Danish‐speaking specialist. Only minimal adjustments were made from the Danish to the Norwegian version. The scoring procedure and symptom definitions follow the Danish BDS checklist (Budtz‐Lilly, Fink, et al. 2015).

#### 2.3.3 Insomnia: Insomnia Severity Index (ISI)

To assess insomnia symptoms over the past 2 weeks, we used the Insomnia Severity Index (ISI) (Morin et al. 2011), which includes 7 items rated from 0 (None/Very satisfied) to 4 (Very/Very dissatisfied). Total scores range from 0 to 28, with higher scores indicating greater severity: 0–7 = no clinically significant insomnia, 8–14 = subthreshold, 15–21 = moderate, and 22–28 = severe. The ISI has high reliability and validity (Bastien et al. 2001), with a Cronbach's α of 0.908 in this cohort.

#### 2.3.4 Fatigue: Chalder Fatigue Questionnaire (CFQ)

To measure physical and mental fatigue, we used the Chalder Fatigue Questionnaire (CFQ) (Chalder et al. 1993), comprising 11 items rated from 0 (Less than usual) to 3 (Much more than usual). Following recommended scoring, responses of 0 or 1 were coded as 0 (non‐case) and 2 or 3 as 1 (case), yielding a total score between 0 and 11. Scores above 4 typically indicate severe fatigue (Jackson 2015). The CFQ is a well‐established, validated measure, also available in Norwegian (Loge et al. 1998), with a Cronbach's α of 0.867 in this cohort.

#### 2.3.5 Catastrophizing: Pain Catastrophizing Scale (PCS)

To evaluate pain‐related catastrophic thinking, we used the Pain Catastrophizing Scale (PCS) (Sullivan et al. 1995), consisting of 13 items rated from 0 (Not at all) to 4 (All the time). Total scores range from 0 to 52, with ≥ 24 indicating clinically relevant catastrophizing and < 15 generally interpreted as non‐catastrophizing. The PCS has good reliability and validity (Sullivan et al. 1998), and the Norwegian version is validated (Fernandes et al. 2012). Cronbach's α in this cohort was 0.946.

#### 2.3.6 Psychological Distress: Hopkins Symptom Checklist‐25 (HSCL‐25)

Psychological distress, including anxiety, depression, and somatization, was measured with the HSCL‐25 (Derogatis et al. 1974), comprising 25 items rated from 1 (Not at all) to 4 (Extremely), yielding a mean score between 1 and 4. A score ≥ 1.75 typically indicates clinically relevant distress (Sandanger, Moum, et al. 1999). The HSCL‐25 has established reliability and validity and is validated in Norwegian (Sandanger, Nygård, et al. 1999). Cronbach's α in this cohort was 0.937.

#### 2.3.7 Perceived Injustice: Injustice Experience Questionnaire (IEQ)

Perceived injustice related to chronic pain was measured with the IEQ (Sullivan et al. 2008), consisting of 12 items rated from 0 (Never) to 4 (All the time), with total scores ranging from 0 to 48. Scores > 19 predict increased pain severity and disability, while ≥ 30 suggest clinical relevance. The IEQ has demonstrated reliability and validity (Rodero et al. 2012; Sullivan et al. 2008) and is validated in Norwegian (Ljosaa et al. 2022). Cronbach's α in this cohort was 0.925.

#### 2.3.8 Health‐Related Quality of Life: EuroQol Five Dimensions Questionnaire (EQ‐5D‐5L)

Health‐related quality of life was measured with the EQ‐5D‐5L (EuroQol Group 1990), comprising five dimensions (mobility, self‐care, usual activities, pain/discomfort, anxiety/depression), each rated on a five‐level scale (1 = no problems to 5 = extreme problems/unable to do). For each participant, the five scores form a health profile (e.g., 1–2–3–2–1), which is converted into a single index value (EQ‐5D Index) using a country‐specific value set, typically ranging from < 0 (worst health states) to 1 (perfect health). The instrument also includes a Visual Analogue Scale (VAS, 0–100) for current health. Both the EQ‐5D Index and the VAS were used as continuous outcomes. As the EQ‐5D‐5L measures distinct domains, internal consistency metrics such as Cronbach's α are not applicable.

#### 2.3.9 Pain‐Related Disability: Oswestry Disability Index (ODI)

Pain‐related disability was measured with a modified ODI (Fairbank and Pynsent 2000), in which references to ‘back’ were removed to broaden applicability. The instrument has 10 items scored 0–5, with a total percentage score from 0% to 100%. Interpretation: 0%–20% = minimal disability, 21%–40% = moderate, 41%–60% = severe, 61%–80% = very severe, 81%–100% = bedbound or exaggerating symptoms. The ODI has demonstrated reliability and validity (Fairbank and Pynsent 2000), and is validated in Norwegian (Grotle et al. 2003). Cronbach's α in this cohort was 0.854.

#### 2.3.10 Patient Global Impression of Change (PGIC)

Overall perceived change following treatment was measured with the PGIC, a single‐item measure at the 12‐month follow‐up. Participants rated changes in symptoms, daily functioning, and quality of life on a 7‐point scale from 1 (Very much better) to 7 (Very much worse), with lower scores indicating greater improvement (Dworkin et al. 2008). The PGIC is brief, widely used in clinical studies, and valued for its simplicity and relevance to patient‐reported outcomes.

#### 2.3.11 Missing Data

Participants who did not respond to at least half of the items in each scale were treated as nonresponders for that scale. For the remaining participants, we calculated the mean score for each scale based on the available items before rescaling the score to the original metric. In all regression analyses, we used listwise deletion.

### 2.4 Ethical Reflections and Data Protection

The authors assert that all procedures contributing to this work comply with the ethical standards of the relevant national and institutional committees on human research and with the Helsinki Declaration of 1975, as revised in 2008. This study is based on anonymized data from a clinical registry and was therefore not subject to review by the Regional Committee for Medical and Health Research Ethics (REK), in accordance with Norwegian regulations. We maintained a strong commitment to ethical practice, and internal ethical procedures were followed to ensure compliance with recognised research standards. All data were collected with explicit informed consent. Participants' rights and autonomy were safeguarded, and robust procedures are in place to ensure data security and confidentiality.

### 2.5 Statistical Analysis

Analyses were conducted in SPSS 29; figures in *ggplot2* (R 4.4.0). Descriptive statistics (frequencies, means, SD) were calculated for the final sample (*N* = 3762). To assess potential selection bias, we compared individuals with valid BDS data (*n* = 3762) to those excluded due to missing BDS checklist responses (*n* = 526) on baseline sociodemographic and clinical variables. Group differences were examined using independent samples *t*‐tests for continuous variables and chi‐square tests for categorical variables.

Although clinical cut‐off values are reported in the description of measures to aid interpretation, all symptom and functioning variables were treated as continuous variables in the statistical analyses.

To answer *the first research question* regarding the prevalence of BDS, we calculated the number and proportion of individuals meeting the criteria for no BDS, moderate BDS (fulfilling 1–3 of five indicators), and severe BDS (fulfilling 4–5 indicators), based on the original five‐indicator case‐finding algorithm, and we used frequency tables and cross‐tabulations. To better understand the structure of symptom indicators, we examined the proportion of individuals who met the threshold within each of the five indicators and reported the distribution of the number of indicators fulfilled (range: 0–5). To ensure that the prevalence of BDS was not solely driven by pain‐related symptoms, we also reported the average number of non‐pain symptoms. We explored BDS severity across sociodemographic subgroups (gender, education, employment) using chi‐square tests.

To address *the second research question* concerning demographic and clinical correlates of BDS, we first presented unstratified baseline characteristics (means, standard deviations, and proportions) for the full sample. We then compared continuous baseline variables across the three BDS severity groups (no BDS, moderate BDS, and severe BDS) using one‐way ANOVAs. To enhance comparability between groups, we also ran ANCOVAs with adjustment for age, gender, education, and employment status. These models provided estimated marginal means and standard errors. Bonferroni correction was applied within each model to account for multiple comparisons, equivalent to a corrected significance threshold of *p* < 0.017. We also conducted additional bivariate and adjusted regression models using the continuous BDS sum score (0–25) as a predictor. We used the same covariates as for the ANCOVAs.

To complement these severity analyses we conducted exploratory comparisons using a diagnostic symptom‐based classification of BDS, namely single‐organ and multi‐organ BDS (Budtz‐Lilly, Fink, et al. 2015; Budtz‐Lilly, Schröder, et al. 2015). Individuals reporting ≥ 4 symptoms within 1–2 clusters or ≥ 4 symptoms distributed across clusters were classified as having single‐organ BDS. Individuals reporting ≥ 4 symptoms in 3–4 clusters were classified as having multi‐organ BDS. These criteria apply to questionnaire‐based assessments and differ from interview‐based diagnostic criteria (Budtz‐Lilly, Fink, et al. 2015; Budtz‐Lilly, Schröder, et al. 2015).

*The third research question* focused on whether baseline BDS symptom burden predicted outcomes at 12‐month follow‐up. We addressed this with adjusted linear regression models with the continuous BDS sum score as the primary predictor. Two separate models were used: For pain‐related disability (ODI), the model was adjusted for baseline ODI and sociodemographic covariates. For perceived global improvement (PGIC), the model included only sociodemographic covariates, since PGIC was not assessed at baseline.

## 3 Results

### 3.1 Descriptive Statistics

The analyses included 3762 individuals with valid BDS data (87.7% of the full cohort). Compared to those excluded, included participants were younger, had longer pain duration, lower disability, higher employment and education levels, but similar gender distribution (see Table S1). Of the included participants, 59.9% were women, the mean age was 48.4 years, 41.3% had higher education, 61.4% were not employed, and pain duration averaged 8.2 years (see Table S2).

### 3.2 Prevalence of BDS

**FIGURE 2:**
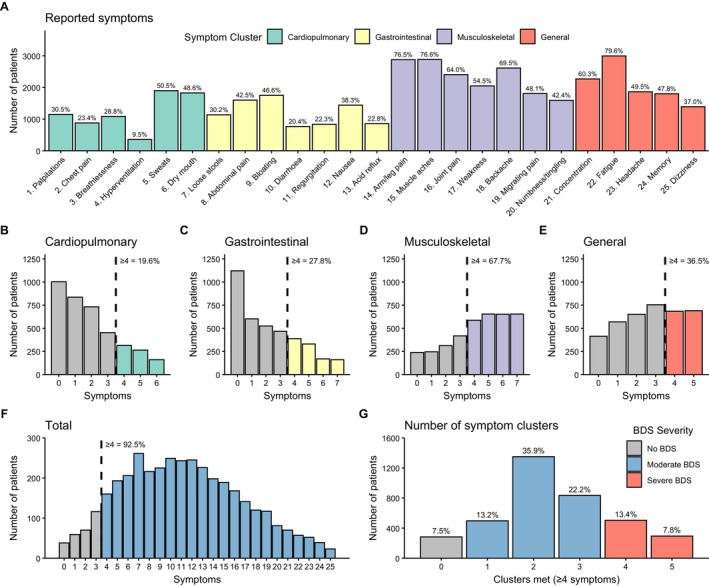
Distribution of bodily distress symptoms across the four symptom clusters. Panel A shows the prevalence of each of the 25 BDS symptoms. Panels B–F show the distribution and proportion of individuals with ≥ 4 symptoms in each symptom cluster (including the total number of symptoms in Panel F). Panel G shows the number of clusters where this criterion was met, defining no BDS, moderate BDS and severe BDS groups.

To address the first research question, we calculated the frequency of each BDS symptom and symptom cluster (Figure 2A–F) and defined BDS severity based on the number of clusters with ≥ 4 symptoms (Figure 2G). In total, 92.5% met criteria for moderate (71.3%) or severe (21.2%) BDS, while 7.5% did not (Table S3). To examine symptom burden beyond pain, we calculated BDS scores excluding the seven pain‐related items. A total of 77.0% reported ≥ 4 non‐pain symptoms (*M* = 7.18, SD = 4.26), supporting a broader symptom profile.

### 3.3 Prevalence of BDS by Sociodemographic Subgroup

**FIGURE 3:**
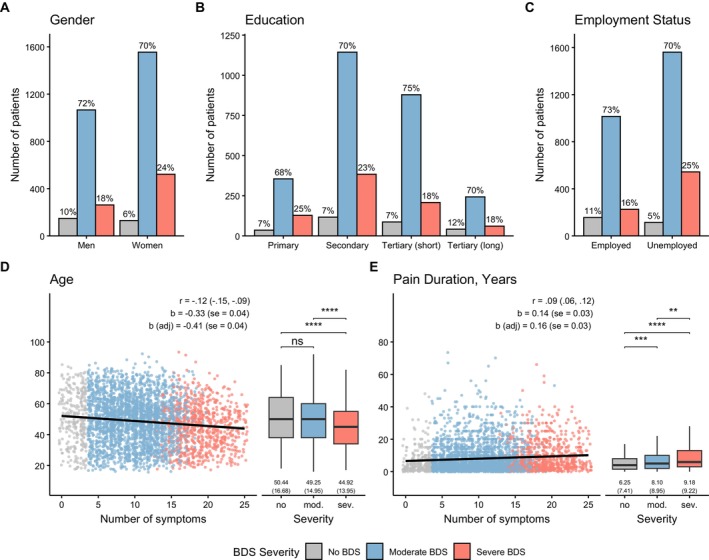
Distribution of gender (A), education (B), employment status (C), age (D) and pain duration (E) across BDS severity groups (No BDS, Moderate BDS and Severe BDS) among the 3762 individuals with valid BDS data. Panels A, B and C shows the distribution of BDS severity classification within each group. Panels D and E shows the association with continuous and categorical BDS and continuous outcomes. Within each panel, results for continuous BDS sum score are presented on the left as scatterplots along with the correlation coefficient (with 95% confidence intervals), the simple regression coefficient (with standard error) and the adjusted regression coefficient (with standard error). The black line is the unadjusted regression slope. The right side of each panel contains results for BDS as categorical severity class, along with unadjusted means and standard deviations for each group. Stars indicate Bonferroni‐adjusted significance tests of mean differences: (^ns^ *p* > 0.05, *p < = 0.05, **p < = 0.01, ***p < = 0.001, ****p < = 0.0001).

BDS severity differed significantly by gender, education, and employment status (all *p* < 0.001; see Figure 3 and Table S4). Severe BDS was more common among women (23.7%), individuals with lower education (24.7% in primary vs. 17.6% in higher tertiary), and those not working (24.5% vs. 16.2%). Severe BDS was associated with longer pain duration and slightly younger age (both *p* < 0.001). No age difference was found between the no and moderate BDS groups (*p* = 0.605).

Exploratory comparisons using the single‐organ and multi‐organ classification yielded the same group distribution as the severity categories, and detailed results are therefore not presented.

### 3.4 Baseline Characteristics by BDS Severity

At baseline, participants reported moderate insomnia, high fatigue, pain catastrophizing, psychological distress, and perceived injustice, along with substantial disability and low quality of life (see Table S5).

**FIGURE 4:**
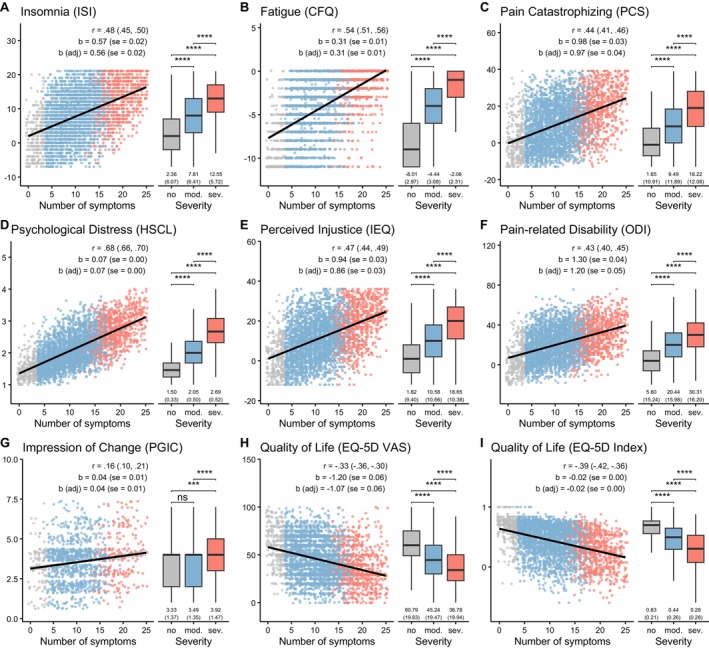
Associations between BDS and clinical variables: (A) insomnia severity (ISI), (B) fatigue (CFQ), (C) pain catastrophizing (PCS), (D) psychological distress (HSCL), (E) perceived injustice (IEQ), (F) pain‐related disability at baseline (ODI), (G and H) quality of life (EQ‐5D) and (I) subjective improvement at 12‐month follow‐up (PGIC). Within each panel, results for continuous BDS sum score are presented on the left as scatterplots along with the correlation coefficient (with 95% confidence intervals), the simple regression coefficient (with standard error) and the adjusted regression coefficient (with standard error). The black line is the unadjusted regression slope. The right side of each panel contains results for BDS as categorical severity class, along with unadjusted means and standard deviations for each group. Stars indicate Bonferroni‐adjusted significance tests of mean differences: (^ns^ *p* > 0.05, *p < = 0.05, **p < = 0.01, ***p < = 0.001, ****p < = 0.0001).

For all variables, more severe BDS was associated with worse symptom profiles and lower quality of life (all *p* < 0.001, Figure 4 and Table S6). Adjusted ANCOVAs confirmed strong associations between BDS severity and all clinical outcomes, with the same severity gradient across groups (all *p* < 0.001; Table 1). Linear regression using the continuous BDS sum score (0–25) showed that higher symptom burden was associated with worse clinical outcomes (Figure 4; Table S7).

**TABLE 1:** Adjusted means (estimated marginal means) of clinical baseline variables by BDS severity level (ANCOVA), adjusted for age, gender, education and employment status. Post hoc comparisons used Bonferroni correction.

Variable	No BDS, *M* (SE)	Moderate BDS, *M* (SE)	Severe BDS, *M* (SE)	*F*(df)	*p*	Post hoc
Insomnia Severity Index (ISI)	9.61 (0.39)	14.89 (0.12)	19.37 (0.23)	*F*(2, 3429) = 263.99	< 0.001	All comparisons: *p* < 0.001
Chalder Fatigue Questionnaire (CFQ)	3.06 (0.18)	6.59 (0.06)	8.88 (0.11)	*F*(2, 3437) = 389.84	< 0.001	All comparisons: *p* < 0.001
Pain Catastrophizing Scale (PCS)	15.01 (0.73)	22.47 (0.24)	30.92 (0.44)	*F*(2, 3401) = 213.36	< 0.001	All comparisons: *p* < 0.001
Hopkins Symptom Check‐List‐25 (HSCL‐25)	1.55 (0.03)	2.06 (0.01)	2.66 (0.02)	*F*(2, 3472) = 623.22	< 0.001	All comparisons: *p* < 0.001
Injustice Experience Questionnaire (IEQ)	14.84 (0.65)	22.74 (0.21)	29.84 (0.38)	*F*(2, 3419) = 226.69	< 0.001	All comparisons: *p* < 0.001
Oswestry Disability Index (ODI)	27.20 (0.93)	40.27 (0.30)	49.64 (0.55)	*F*(2, 3472) = 230.26	< 0.001	All comparisons: *p* < 0.001
EQ‐5D Visual Analogue Scale (VAS)	59.47 (1.19)	45.14 (0.38)	37.78 (0.71)	*F*(2, 3427) = 123.03	< 0.001	All comparisons: *p* < 0.001
EQ‐5D Index	0.62 (0.02)	0.44 (0.01)	0.30 (0.01)	*F*(2, 3478) = 176.53	< 0.001	All comparisons: *p* < 0.001

### 3.5 Prediction of 12‐Month Outcomes From Baseline BDS Sum Score

Higher baseline BDS sum scores predicted greater pain‐related disability (adjusted for baseline ODI) and poorer global improvement at 12 months (both *p* < 0.001; Table 2).

**TABLE 2:** Multiple linear regression results predicting 12‐month outcomes from baseline BDS sum score (Range 0–25), adjusted for baseline ODI (only ODI‐model) and demographic.

Outcome	Predictor	B	SE	*β*	95% CI for B	*p*
ODI (12‐month)	BDS sum score	0.29	0.07	0.09	(0.15–0.43)	< 0.001
	Baseline ODI	0.74	0.02	0.67	(0.69–0.78)	< 0.001
	Age	−0.02	0.03	−0.02	(−0.08–0.03)	0.353
	Gender	−0.64	0.71	−0.02	(−2.04–0.75)	0.368
	Education	−0.71	0.43	−0.03	(−1.56–0.13)	0.099
	Employment status	4.14	0.78	0.11	(2.61–5.68)	< 0.001
PGIC (12‐month)	BDS sum score	0.04	0.01	0.14	(0.02–0.05)	< 0.001
	Age	0.01	0.00	0.06	(0.00–0.01)	0.049
	Gender	−0.10	0.08	−0.04	(−0.26–0.06)	0.223
	Education	−0.10	0.05	−0.06	(−0.20 to −0.01)	0.040
	Employment status	0.32	0.09	0.11	(0.15–0.49)	< 0.001

## 4 Discussion

This study examined the prevalence, symptom structure and prognostic significance of BDS among individuals referred to a tertiary pain clinic. We evaluated BDS both categorically and dimensionally, which enabled analyses across diagnostic thresholds and the full distribution of symptom burden.

In total, 92.5% met criteria for moderate (71.3%) or severe (21.2%) BDS, compared to 11.8 to 16.1% in the general population (Häuser et al. 2020; Petersen, Schröder, et al. 2020) and 26.8% in somatic outpatient clinics (Ma et al. 2022). Our sample was drawn from a tertiary, highly specialised pain clinic where referrals are largely restricted to patients with long‐standing, treatment‐resistant and diagnostically complex presentations. These rates therefore likely reflect referral patterns and the selection of individuals with complex symptom profiles (Landmark et al. 2024) rather than diagnostic inflation (Budtz‐Lilly, Fink, et al. 2015; Petersen, Rosendal, et al. 2020). The near‐universal presence of BDS indicates that tertiary pain clinics primarily manage complex, multisystem distress rather than isolated pain conditions (Gormsen et al. 2025).

### 4.1 Differences Between BDS, BDD, and FSD

In this study, BDS severity reflects a symptom pattern derived from the BDS checklist and does not represent a clinical diagnosis. This operationalisation follows the multisystem symptom core emphasised in the Functional Somatic Disorder framework (Burton et al. 2020) and remains distinct from ICD‐11 Bodily Distress Disorder (BDD) (Henningsen and Löwe 2025). BDD is included in the section of mental disorders and requires persistent bodily symptoms together with psychobehavioural features such as excessive worry, avoidance of activity and persistent catastrophic interpretations. The absence of such criteria in BDS allows clinicians in primary care to use it as a pragmatic case finding construct without assigning a mental disorder diagnosis (Häuser et al. 2025).

Our findings show both a high prevalence of multisystem symptom patterns and a clear severity gradient. These features align with the Functional Somatic Disorder framework (Burton et al. 2020). This framework brings together lumper and splitter perspectives by allowing conditions such as fibromyalgia and CFS/ME to be classified both within organ‐based and within broader transdiagnostic categories. Bodily distress can be seen as existing on a continuum, yet BDS was developed as a categorical unifying diagnosis with single‐organ and multisystem subtypes, providing an alternative to syndrome‐specific classifications (Fink and Schröder 2010). In this model, BDS captures empirically derived symptom patterns that extend beyond organ‐based syndrome labels. This reflects a multimorbidity profile rather than several distinct co‐occurring syndromes. Recent work suggests that this broader perspective is particularly relevant in pain populations, where overlapping symptom patterns are common (Gormsen et al. 2025). Overall, our findings contribute to the ongoing debate on how persistent physical symptoms should be classified (Henningsen et al. 2019; Rief et al. 2019).

### 4.2 BDS as Categorical Versus Dimensional

Higher BDS symptom burden was associated with more severe insomnia and fatigue, elevated psychological distress, and greater functional disability. These associations were consistent across both categorical and dimensional approaches, supporting BDS as an index of clinical severity. The severity gradient suggests that BDS captures graded rather than discrete differences in multisystem symptom burden. This interpretation aligns with neurobiological and cognitive models linking persistent somatic distress to long‐term dysregulation in autonomic, interoceptive and predictive systems (Henningsen, Gündel, et al. 2018), where maladaptive attentional and interpretive patterns may reinforce symptom perception over time. Supplementary subtype analyses showed a similar pattern across single‐organ and multi‐organ BDS, suggesting that overall symptom burden may be more informative than cluster distribution. Previous studies have documented substantial commonalities between BDS and other multisystem symptom conditions, including CFS/ME and primary pain syndromes such as fibromyalgia (Fink and Schröder 2010), supporting a dimensional perspective in which symptom severity and functional impact exist along a continuum. This perspective may also allow treatments to be guided more directly by symptom burden rather than diagnostic labels.

### 4.3 BDS and Clinical Prognosis

Severe BDS predicted greater pain‐related disability and lower perceived global improvement at 12‐month follow‐up, even after adjustment for demographic and baseline clinical variables. To our knowledge, no previous studies have demonstrated that BDS severity predicts long‐term outcomes in tertiary pain care. Although psychological distress is a known prognostic factor, BDS may represent a broader, clinically relevant indicator of overall symptom complexity, which is often overlooked when assessments focus narrowly on pain.

### 4.4 Clinical Implications

Compared to syndrome specific diagnoses, BDS may direct attention beyond the pain complaint to the broader constellation of somatic symptoms that often accompany chronic pain, including gastrointestinal and autonomic symptoms. Even in multidisciplinary pain clinics, interventions are typically organised around pain, while other symptoms are referred elsewhere. A BDS framework may therefore support a more integrated focus on shared maintaining processes, including fear‐driven expectations, avoidance, and psychological inflexibility. Patients also tend to narrow their focus when consultations centre exclusively on pain, yet our data indicate substantial multisystem burden.

Group‐based Acceptance and Commitment Therapy has shown promising effects in individuals with BDS (Kallesøe et al. 2021), but the added value of BDS lies in broadening the clinical focus beyond pain and supporting the targeted use of integrative approaches, including newer mind–body interventions relevant for nociplastic conditions. This broader focus could provide interdisciplinary pain teams with a clearer rationale for treatment planning and for prioritising interventions across disciplines. Early identification of multisystem burden can enhance triage and support more coherent interdisciplinary care. The BDS checklist, validated across general population, primary care and specialist settings (Petersen, Rosendal, et al. 2020), provides a practical tool for early symptom mapping that helps clinicians identify multisystem burdens and deliver more timely targeted interventions. This is consistent with recent recommendations for integrated care in high‐burden cases (Sattel et al. 2023).

Current guidelines differ markedly across syndromes, with some emphasising activity and others recommending energy conservation strategies. For individuals with broad and overlapping symptom patterns, such divergent recommendations create fragmented clinical pathways. Care pathways are largely organised around syndrome‐specific diagnoses, yet individuals with complex multisystem symptoms often do not fit these structures. The NICE guidelines for chronic primary pain represent a more multidisciplinary and psychologically informed approach (National Institute for Health and Care Excellence 2021a), but this stands in contrast to narrower syndrome‐specific recommendations in related conditions (National Institute for Health and Care Excellence 2021b). Inconsistencies across diagnostic guidelines may therefore hinder coherent care for individuals presenting with complex and overlapping symptom profiles.

### 4.5 Strengths and Limitations

Key strengths of this study include the large sample, the use of validated measures and the 12‐month follow‐up, which provides robust evidence for both cross‐sectional and longitudinal associations. Because both predictors and outcomes were based on self‐report, some of the observed associations may reflect general response tendencies rather than actual changes in symptoms or functioning, and future studies should therefore include clinician‐rated assessments or objective indicators of functioning. BDS classification was also based on self‐report rather than structured clinical interviews, which may have affected prevalence estimates (Petersen et al. 2021). Missing data, particularly in the BDS checklist, may have contributed to selection bias. Participants excluded because of incomplete BDS data reported higher disability, suggesting that overall symptom burden in the analysed sample may have been underestimated. Finally, as with all observational designs, causal inference is limited, and it cannot be determined whether poorer outcomes among individuals meeting BDS criteria reflect severity, shared mechanisms or inadequate treatment.

## 5 Conclusion

BDS was highly prevalent in this tertiary pain clinic and showed strong associations with more severe symptoms and poorer long‐term outcomes. The clear severity gradient supports a dimensional conceptualization that moves beyond a single‐symptom focus (e.g., pain alone) and traditional diagnostic classifications. BDS may provide a clinically meaningful framework for identifying symptom complexity and strengthening interdisciplinary care for persistent symptoms more broadly.

## Author Contributions

S.E.R. and L.L. designed the study. L.L. and H.F.S. analysed the data, and all authors critically examined the results. L.L. had the primary responsibility for drafting the manuscript, which was revised by H.F.S., E.A.F., L.E.O.K. and S.E.R. H.F.S. prepared the figures. All authors discussed the results, contributed to the writing, approved the final version of the manuscript, and agree to be accountable for all aspects of the work.

## Funding

The research received no specific grant from any funding agency, commercial or not‐for‐profit sectors. L.L.'s position is funded by the Municipalities in Viken, Norway, and the Research Council of Norway. H.F.S.'s contribution is funded by the Mind‐Body Lab at the University of Oslo and partly supported by the Research Council of Norway through its Centers of Excellence funding scheme (#262700).

## Conflicts of Interest

L.L. and S.E.R. receive honoraria for lectures and workshops on stress, persistent physical symptoms and coping. L.L. is also a member of Athenas.no, a speaker bureau. The other authors declare no conflicts of interest.

## Supporting information


**Tables S1–S7:** ejp70212‐sup‐0001‐TableS1‐S7.docx.

## Data Availability

The dataset used in this study originates from the Oslo Pain Registry (OPR). Access to the data is subject to approval by the OPR board. Inquiries regarding data access should be directed to chrekh@ous-hf.no. Analytical code transparency and research materials transparency: All statistical analyses were conducted using SPSS version 29. Figures were produced using ggplot2 in R version 4.4.0. Analytical code for figure generation is available from the authors upon reasonable request.
