# Supplementary material for: Prevalence of Bodily Distress Syndrome and Prediction of Patient Outcomes: Cohort Study of 3762 Individuals With Persistent Pain
Source: Eur J Pain. 2026 Jan 20;30(2):e70212. doi: 10.1002/ejp.70212 (PMC12818827; doi:10.1002/ejp.70212)
Supplement: Supplementary file 1 — Tables S1–S7: ejp70212‐sup‐0001‐TableS1‐S7.docx. [file EJP-30-0-s001.docx]

# SUPPLEMENTARY

**Supplementary tableS1**. Comparison of included vs. excluded participants

| **Variable** | **Included (n=3762)** | **Excluded (n=526)** | **p-value** |
| --- | --- | --- | --- |
| Age (mean, SD) | 48.42 (14.99) | 57.28 (17.43) | <.001 |
| Gender: Female (%) | 59.9% (n=2207/3683) | 57.1% (n=283/496) | 0.222 |
| Education: |  |  |  |
| Primary (%) | 14.1% (n=519/3684) | 25.9% (n=124/479) | <.001 |
| Secondary (%) | 44.7% (n=1645/3684) | 42.2% (n=202/479) | <.001 |
| Tertiary (short) (%) | 31.9% (n=1174/3684) | 26.9% (n=129/479) | <.001 |
| Tertiary (long) (%) | 9.4% (n=346/3684) | 5.0% (n=24/479) | <.001 |
| Employment: Working (%) | 38.6% (n=1397/3617) | 21.0% (n=99/471) | <.001 |
| Pain duration (years, mean, SD) | 8.19 (8.93) | 1.70 (4.90) | <.001 |
| Baseline ODI (mean, SD) | 41.43 (17.07; n=3752) | 51.89 (17.61; n=500) | <.001 |

**Supplementary tableS2**. Sociodemographic characteristics and prevalence of BDS classification (*N* = 3762)

| **Variable** | **Category** | **n** | **%** |
| --- | --- | --- | --- |
| Gender | Men | 1476 | 40.1 |
|  | Women | 2207 | 59.9 |
|  | Missing | 79 | 2.1^1^ |
| Education level | Primary and lower secondary education (1-10 years) | 519 | 14.1 |
|  | Upper secondary education  (11-13 years) | 1645 | 44.7 |
|  | Lower tertiary education  (14-17 years) | 1174 | 31.9 |
|  | Higher tertiary education  (>17 years) | 346 | 9.4 |
|  | Missing | 78 | 2.1^1^ |
| Work status | Working/student/ military service | 1397 | 38.6 |
|  | Not working | 2220 | 61.4 |
|  | Missing | 145 | 3.9^1^ |
| BDS severity class | No BDS | 283 | 7.5 |
|  | Moderate BDS | 2681 | 71.3 |
|  | Severe BDS | 798 | 21.2 |
| Continuous variable | n | Mean | SD |
| Age | 3762 | 48.42 | 14.99 |
| Pain duration | 3762 | 8.19 | 8.93 |

¹Percentage of total N (3762).

**Supplementary tableS3.** Distribution of patients meeting the criterion of ≥4 symptoms within each BDS cluster and cumulative number of clusters meeting this threshold

| **Symptom cluster** | **N (%) with 0 symptoms** | **N (%) with 1 symptom** | **N (%) with 2 symptoms** | **N (%) with 3 symptoms** | **N (%) with 4+ symptoms** |
| --- | --- | --- | --- | --- | --- |
| Cardiopulmonary | 1003 (26.7%) | 836 (22.2%) | 732 (19.5%) | 453 (12.0%) | 738 (19.6%) |
| Gastrointestinal | 1121 (29.8%) | 602 (16.0%) | 525 (14.0%) | 467 (12.4%) | 1047 (27.8%) |
| Musculoskeletal | 239 (6.4%) | 246 (6.5%) | 312 (8.3%) | 418 (11.1%) | 2547 (67.7%) |
| General | 414 (11.0%) | 569 (15.1%) | 651 (17.3%) | 754 (20.0%) | 1374 (36.5%) |
| Total clusters (0-4+) | 283 (7.5%) | 497 (13.2%) | 1350 (35.9%) | 834 (22.2%) | 798 (21.2%) |

**Supplementary tableS4**. Sociodemographic distribution by BDS severity level (*N* = 3762)

| **Variable** | **No BDS % (n)** | **Moderate BDS % (n)** | **Severe BDS % (n)** | **Total n** |
| --- | --- | --- | --- | --- |
| Gender |  |  |  |  |
| Men | 10.0% (n=148) | 72.2 % (n=1066) | 17.8% (n=262) | 100.0% (n=1476) |
| Women | 5.9% (n=130) | 70.5 % (n=1555) | 23.7% (n=522) | 100% (n=2207) |
| Education level |  |  |  |  |
| Primary and lower secondary education (1-10 years) | 6.9% (n=36) | 68.4% (n=355) | 24.7% (n=128) | 100.0% (n=519) |
| Upper secondary education (11-13 years) | 7.1% (n=117) | 69.5% (n=1144) | 23.3% (n=384) | 100.0% (n=1645) |
| Lower tertiary education (14-17 years) | 7.4% (n=87) | 74.9% (n=879) | 17.7% (n=208) | 100% (n=1174) |
| Higher tertiary education (>17 years) | 12.1% (n=42) | 70.2% (n=243) | 17.6% (n=61) | 100.0% (n=346) |
| Work or life situation |  |  |  |  |
| Working/student/military service | 11.2% (n=156) | 72.7% (n=1015) | 16.2% (n=226) | 100.0% (n=1397) |
| Not working | 5.2% (n=115) | 70.3% (n=1561) | 24.5% (n=544) | 100.0% (n=2220) |

**Note:** Percentages are column-wise and indicate the distribution of each sociodemographic characteristic within each BDS severity group.

**Supplementary tableS5.** Baseline clinical characteristics

| **Variables** | **N** | **Mean** | **SD** |
| --- | --- | --- | --- |
| Sleep |  |  |  |
| Insomnia Severity Index (ISI, Range: 0-28) | 3703 | 15.40 | (6.75) |
| Fatigue |  |  |  |
| Chalder Fatigue Questionnaire (CFQ, Range: 0-11) | 3715 | 6.80 | (3.26) |
| Pain catastrophizing |  |  |  |
| Pain Catastrophizing Scale (PCS, Range: 0-52) | 3674 | 23.74 | (12.64) |
| Psychological distress |  |  |  |
| Hopkins Symptom Check-List-25 (HSCL-25, Range: 1-4) | 3755 | 2.15 | (0.58) |
| Perceived injustice |  |  |  |
| Injustice Experience Questionnaire (IEQ; Range: 0-48) | 3697 | 23.65 | (11.35) |
| Pain-related disability |  |  |  |
| Oswestry Disability Index (ODI, Range: 0-100) | 3752 | 41.43 | (17.07) |
| General health and quality of life |  |  |  |
| Your Health (VAS) | 3700 | 44.59 | (20.41) |
| EQ-5D Index | 3762 | 0.42 | (0.28) |

**Supplementary tableS6**. Descriptive statistics and unadjusted group differences for continuous variables across BDS severity levels (No BDS, moderate BDS, severe BDS)

| **Variable** | **No BDS**  **M (SD)** | **Moderate BDS**  **M (SD)** | **Severe BDS**  **M (SD)** | **F (df1, df2)** | **p-value** | **Post hoc** |
| --- | --- | --- | --- | --- | --- | --- |
| Age | 50.44 (16.68) | 49.25 (14.95) | 44.92 (13.95) | F(2, 3759) = 28.79 | <.001 | Severe BDS > No BDS and Severe BDS > Moderate BDS (both p < .001); No BDS vs Moderate BDS (p = .605) |
| Pain duration (years) | 6.25 (7.41) | 8.10 (8.95) | 9.18 (9.22) | F(2, 3759) = 11.80 | <.001 | Moderate BDS > No BDS (p = .003); Severe BDS > No BDS (p < .001) and Severe > Moderate ( p = .008) |
| Insomnia Severity Index (ISI) | 9.36 (6.07) | 14.81 (6.41) | 19.55 (5.72) | F(2, 3700) = 314.81 | <.001 | Moderate > No BDS (p < .001); Severe > No BDS and Severe > Moderate (both p < .001) |
| Chalder Fatigue Questionnaire (CFQ) | 2.99 (2.97) | 6.56 (3.08) | 8.94 (2.31) | F(2, 3712) = 454.79 | <.001 | Moderate > No BDS (p < .001); Severe > No BDS and Severe > Moderate (both p < .001) |
| Pain Catastrophizing Scale (PCS) | 14.65 (10.92) | 22.49 (11.89) | 31.22 (12.08) | F(2, 3671) = 250.66 | <.001 | Severe > Moderate > No BDS (all p < .001) |
| Hopkins Symptom Checklist-25 (HSCL-25) | 1.50 (0.33) | 2.05 (0.50) | 2.69 (0.52) | F(2, 3752) = 779.38 | <.001 | Moderate > No BDS (p < .001); Severe > No BDS and Severe > Moderate (both p < .001) |
| Injustice Experience Questionnaire (IEQ) | 13.82 (9.40) | 22.58 (10.66) | 30.65 (10.38) | F(2, 3694) = 309.57 | <.001 | Moderate > No BDS (p < .001); Severe > No BDS and Severe > Moderate (both p < .001) |
| Oswestry Disability Index (ODI) | 25.60 (15.24) | 40.44 (15.99) | 50.31 (16.20) | F(2, 3749) = 266.25 | <.001 | Moderate > No BDS (p < .001); Severe > No BDS and Severe > Moderate (both p < .001) |
| Your Health (VAS) | 60.79 (19.83) | 45.24 (19.47) | 36.78 (19.94) | F(2, 3697) = 158.00 | <.001 | No BDS > Moderate (p < .001); No BDS > Severe and Moderate > Severe (both p < .001) |
| EQ-5D Index | 0.63 (0.21) | 0.44 (0.26) | 0.28 (0.28) | F(2, 3759) = 220.77 | <.001 | No BDS > Moderate (p < .001); No BDS > Severe (p < .001); Moderate > Severe (p < .001) |

*Values represent *mean (standard deviation)*. Higher scores reflect greater symptom severity or functional impairment, except for the EQ-5D Index and EQ VAS, where higher scores indicate better health status.

**Supplementary tableS7**. Linear regression models predicting baseline symptom and function measures from continuous BDS sum score (range 0–25)

| **Variable** | **B** | **SE** | **β** | **95% CI for B** | **p** |
| --- | --- | --- | --- | --- | --- |
| Insomnia Severity Index (ISI) | 0.57 | 0.02 | 0.48 | 0.54–0.61 | <.001 |
| Chalder Fatigue Questionnaire (CFQ) | 0.31 | 0.01 | 0.54 | 0.30–0.33 | <.001 |
| Pain Catastrophizing Scale (PCS) | 0.98 | 0.03 | 0.44 | 0.92–1.05 | <.001 |
| Hopkins Symptom Check-List-25 (HSCL-25) | 0.07 | 0.00 | 0.68 | 0.07–0.07 | <.001 |
| Injustice Experience Questionnaire (IEQ) | 0.94 | 0.03 | 0.47 | 0.89–1.00 | <.001 |
| Oswestry Disability Index (ODI) | 1.30 | 0.05 | 0.43 | 1.21–1.39 | <.001 |
| EQ-5D Visual Analogue Scale (VAS) | -1.20 | 0.06 | -0.33 | -1.31– -1.09 | <.001 |
| EQ5D Index | -0.02 | 0.00 | -0.39 | -0.02– -0.02 | <.001 |

* Higher BDS sum score indicates greater bodily symptom burden, except for the EQ-5D Index and EQ VAS, where higher scores reflect better health. Models treat BDS sum score as a continuous predictor.
